# Mature beyond their years: young children who escape detection of parasitemia despite living in settings of intense malaria transmission

**DOI:** 10.1042/BST20230401

**Published:** 2024-06-26

**Authors:** Prasida Holla, Jyoti Bhardwaj, Tuan M. Tran

**Affiliations:** 1 Ryan White Center for Global Health and Pediatric Infectious Diseases, Department of Pediatrics, Indiana University School of Medicine, Indianapolis, IN 46202, U.S.A.;; 2 Division of Infectious Diseases, Department of Medicine, Indiana University School of Medicine, Indianapolis, IN 46202, U.S.A.;; 3 Department of Microbiology and Immunology, Indiana University School of Medicine, Indianapolis, IN 46202, U.S.A.

## Abstract

Despite having the highest risk of progressing to severe disease due to lack of acquired immunity, the youngest children living in areas of highly intense malaria transmission have long been observed to be infected at lower rates than older children. Whether this observation is due to reduced exposure to infectious mosquito bites from behavioral and biological factors, maternally transferred immunity, genetic factors, or enhanced innate immunity in the young child has intrigued malaria researchers for over half a century. Recent evidence suggests that maternally transferred immunity may be limited to early infancy and that the young child’s own immune system may contribute to control of malarial symptoms early in life and prior to the development of more effective adaptive immunity. Prospective studies of active and passive detection of *Plasmodium falciparum* blood-stage infections have identified young children (<5 years old) who remain uninfected through a defined surveillance period despite living in settings of highly intense malaria transmission. Yet, little is known about the potential immunological basis for this ‘aparasitemic’ phenotype. In this review, we summarize the observational evidence for this phenotype in field studies and examine potential reasons why these children escape detection of parasitemia, covering factors that are either extrinsic or intrinsic to their developing immune system. We discuss the challenges of distinguishing malaria protection from lack of malaria exposure in field studies. We also identify gaps in our knowledge regarding cellular immunity in the youngest age group and propose directions that researchers may take to address these gaps.

## Introduction

**Figure 1.**
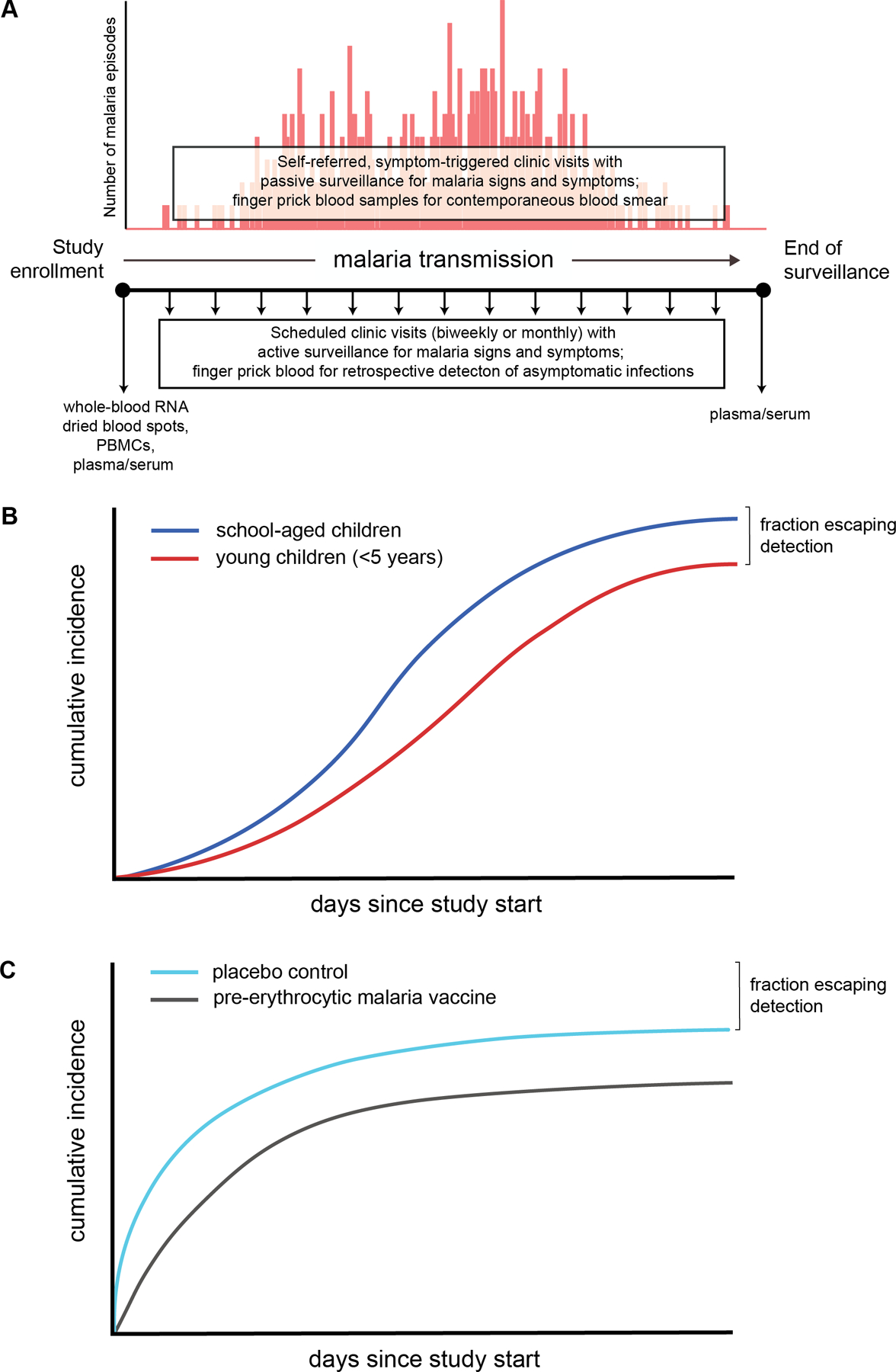
Surveillance of malaria infections in prospective cohort studies. (**A**) Design of a prototypical prospective cohort study with passive and active detection of malaria episodes and *P. falciparum* infections. Figure adapted from [11]. Participant blood samples are collected at the start of the study to allow biological assessments that can be linked to prospective risk of clinical malaria or *P. falciparum* infection. Plasma/serum collected at the end of the surveillance period can be used to evaluate seroconversion of *P. falciparum* stage-specific antibodies as a surrogate of malaria exposure. PBMCs, peripheral blood mononuclear cells. Hypothetical infection cumulative incidence plots showing the fraction of young children escaping parasite detection for (**B**) an observational cohort study of natural *P. falciparum* infections in children and (**C**) a pre-erythrocytic malaria vaccine trial in infants aged 5–12 months. These conceptual plots were modeled after unpublished and published data from the Kalifabougou cohort study [10] and the KSPZV1 malaria vaccine trial [12,13], respectively.

Malaria, caused by *Plasmodium* parasites that are transmitted to humans by *Anopheles* mosquitos, affects over 230 million people globally each year, resulting in over 600 000 deaths [1]. The vast majority of these deaths occur in African children under 5 years of age suffering from severe *Plasmodium falciparum* malaria [1]. Despite having the highest risk of progressing to severe disease due to lack of acquired immunity, the youngest children living in areas of high malaria transmission have long been observed to be infected at lower rates than older children [2–4]. The factors underlying the reduced prevalence of malaria infection observed in children with immature and inexperienced immune systems have intrigued malaria researchers for decades [2,4–7]. From an evolutionary perspective, enhanced innate protection against malaria infection early in life, even if imperfect and short-lived, would still reduce the total number of potentially life-threatening malaria episodes prior to the acquisition of more effective adaptive immunity, especially in rural settings in sub-Saharan Africa where entomological inoculation rates (EIRs) average 144 infective bites per person per year [8]. The increased prevalence of symptomatic malaria, which prompts treatment and parasite clearance by anti-malarials, among the youngest children may partially explain reduced infection prevalence observed in cross-sectional studies [9,10]. However, other explanations that are extrinsic or intrinsic to the young host have been postulated as contributing to these observations [2,5,6]. Longitudinal cohort studies conducted in malaria-endemic communities have sought to identify predictors of sterile or clinical protection by correlating specific factors measured at baseline to prospective risk of *Plasmodium* infection or symptomatic malaria episodes, respectively (Figure 1A). These studies employ active and passive case detection, and protection is measured as either the absence of events or a delay in time to event. Depending on the study objectives, an event can be either detectable parasitemia (identified by blood smear or PCR) or clinical malaria (parasitemia above a pre-defined density threshold plus malarial symptoms, usually documented fever). Thus, malaria risk can be reported in terms of binary outcomes (absence or occurrence of any infection or clinical malaria episode during the surveillance period) or as continuous variables (time-to-infection or time-to-clinical-malaria). Interestingly, prospective cohort studies that assess time-to-infection have shown that a fraction of young children remain parasite negative during a defined surveillance period despite high malaria transmission [10,12,14,15]. Given the lack of evidence for naturally acquired immunity to *P. falciparum* infection even as immunity to clinical malaria is clearly acquired in individuals who experience years of intense transmission [10], one possible explanation is that elite innate immunity early in life either prevents liver-stage infection or controls blood-stage parasitemia to undetectable levels. Yet little is known about the cellular immune responses that may contribute to the apparent resistance to malaria infection in these children. In this review, we summarize the evidence to date for a subset of young children living in settings of intense malaria transmission with an ‘aparasitemic’ phenotype, which we define as the continued absence of detectable *P. falciparum* parasites in the blood despite active longitudinal surveillance for asexual blood-stage infection during a specified time period. We examine potential reasons why these children escape detectable parasitemia, covering factors extrinsic and intrinsic to their developing immune system and identifying gaps in our knowledge regarding cellular immunity in this population. We also discuss the challenges of distinguishing protection from detectable infection or clinical malaria from lack of malaria exposure in field studies and how researchers may address this.

## Decreased malaria infection rates in youngest children (<5 years)

Infants and young children have been observed to have decreased rates of *P. falciparum* infection by blood smear relative to older children up to 15 years of age [2–4,9,16–19]. Studies that used more sensitive molecular parasite detection have also found decreased parasite prevalence in the youngest children when compared with school-aged children [16–18,20,21]. Such differences appear to be most pronounced in areas of high malaria transmission intensity [22]. These cross-sectional community prevalence studies have generally captured pre-symptomatic and truly asymptomatic malaria infections. Thus, the increased prevalence in school-aged children reflects, to a large degree, acquisition of clinical immunity to parasitemia after several years cumulative malaria exposure [10,23]. However, period prevalence and incidence rates measured by rapid diagnostic tests (RDT) in treatment-seeking, febrile patients at health facilities on the Kenyan coast, where overall RDT infection prevalence was 9.9%, also increased with age from infancy to ~9 years of age before decreasing towards adulthood [24]. Evidence for decreased malaria infection rates in the youngest children can also be found in longitudinal cohorts. A 500-participant cohort study in an area of Mali where malaria transmission is seasonal and intense showed that the 1-year longitudinal prevalence of *P. falciparum*, measured by PCR, was highest in individuals aged 9–16 years when compared with those aged 1–4 years or >17 years [25]. In a longitudinal study of 114 Malawian children living in an area of intense perennial transmission, children <5 years of age had fewer incident *P. falciparum* infections by PCR per child relative to children aged 5–15 years [26]. Even within a narrow age range, infants aged 6–11 months appear to be more protected than older children aged 12–24 months in areas of Uganda with high malaria incidence rates (6.41 vs. 7.24 cases per person-years at risk, respectively) [27]. Thus, there is considerable evidence that, in areas of high malaria endemicity, the youngest children are infected at lower rates when compared with older children despite having relatively less acquired immunity to the parasite.

## An ‘aparasitemic’ phenotype in infants and young children

Further evidence for decreased rates of incident *P. falciparum* clinical episodes and infection in infants and young children can be found in prospective studies of both active and passive malaria surveillance in high malaria transmission settings. In a longitudinal study in Kenya, more children were observed to have no clinical malaria episodes during a 5-year surveillance period than what otherwise would be expected based on a fitted Poisson distribution, particularly among children <5 years of age [28]. In a prospective cohort study of children and adults conducted in Mali, *P. falciparum* infections were determined over the course of a 6-month malaria transmission season by retrospective PCR analysis of dried blood spots collected every 2 weeks and at sick visits [10]. The main finding of this study was that the infection risk did not decrease with increasing years of malaria exposure, providing evidence that naturally acquired sterile immunity to *P. falciparum* does not occur even in high malaria transmission settings. Interestingly, 10% of children in the youngest age group (4–6 years) in the published study remained uninfected at the end of 6 months of intense malaria transmission compared with <5% in the older age groups [10]. In unpublished data from the same year of the cohort, ~25% of children aged 3 months to 3 years remained uninfected during the first year of intensive surveillance but eventually had detectable parasitemia during the ensuing malaria seasons (Peter Crompton, personal communication, conceptualized in Figure 1B). A longitudinal study of 264 Papua New Guinean children aged 0.9–3.2 years reported that 13% remained free of PCR-detectable *P. falciparum* infections despite up to 69 weeks of follow-up that included active surveillance for malaria morbidity every 2 weeks and infections every 8–9 weeks as well as passive malaria case detection [29]. A longitudinal birth cohort study in a high malaria transmission area of Tanzania reported 63 children aged 1–3.5 years (9.2% of the cohort) who had no blood smear-positive slides despite 2 years of biweekly active parasitological surveillance [15]. In a study of Ugandan children residing in an area of high transmission intensity, a subset of 16 children for whom longitudinal infection histories were shown through their first 5 years of life, two children experienced infection-free periods of at least ~18 months despite surveillance by monthly blood smears and symptom-triggered visits [30]. Additional evidence can be found in the control arms of malaria vaccine studies. In a phase 2b trial of the pre-erythrocytic vaccine RTS,S/AS02 conducted in Mozambican children aged 1–4 years (‘cohort 2’), of the 146 children in the control arm who completed the trial, 10 (6.8%) remained free of parasitemia during 6 months of intensive surveillance that consisted of at least monthly blood smears and passive surveillance [14]. More recently, a clinical trial of the radiation-attenuated *P. falciparum* sporozoite (PfSPZ) vaccine conducted in Kenyan infants aged 5–12 months, ~34% of infants in the normal saline placebo arm who completed the protocol remained uninfected despite 6 months of monthly blood smears and passive surveillance (conceptualized in Figure 1C) [12,13]. That such a high proportion of infants remained ‘aparasitemic’ is made more remarkable by the highly intense malaria transmission that occurs in Siaya county, where the trial was conducted, with EIR reaching as high as 29.9 infective bites per person per month during peak transmission [31]. These studies suggest that a subset of infants and young children somehow escape detectable parasitemia for a finite time period despite living in areas of highly intense malaria transmission.

## Potential explanations extrinsic to the developing immune system

Numerous explanations have been proposed for the observation of reduced infection in infants and young children. Behavioral measures such as swaddling of infants and increased used of insecticide-treated nets for the youngest children may reduce exposure to infectious mosquito bites [32]. Young children are bitten by mosquitoes less frequently than older children and adults, a finding that has been correlated to body surface area [19,33,34]. Fetal hemoglobin has been suggested as being malaria protective based on *in vitro* experiments [35,36] and a modest association with protection in the youngest children [37], especially in presence of hemoglobin AA [38]. Host genetic variants, especially erythrocyte polymorphisms and hemoglobinopathies, can confer protection against malaria [39]. However, little evidence exists for protective synergy between younger age and host polymorphisms. Although sickle cell trait (HbAS) protects against all-cause mortality, severe malarial anemia, and high-density parasitemia in the highest risk group of young children aged 2–16 months [40], the protection afforded by HbAS against molecular force of *P. falciparum* infection and mild clinical malaria actually increases with age up to 8–10 years [41,42].

Placentally transferred maternal anti-malarial IgG antibodies have been proposed as one mechanism to explain malaria-resistance in infants [5]. However, the evidence that maternal antibodies protect against malaria infection and clinical malaria has been mixed and may be dependent on the specific malaria antigen and IgG subclass [6]. For example, neither IgM nor IgG antibodies specific for pre-erythrocytic and erythrocytic antigens were associated with time-to-first *P. falciparum* infection or the number of infections during the first year of life in a longitudinal study of Cameroonian infants [43]. However, a birth cohort study in Burkina Faso showed that high level maternal IgG to *P. falciparum* erythrocytic antigens EBA140, EBA175, MSP1_42_, and MSP5 independently predicted protection from clinical malaria during the first year of life [44]. Notably, any malaria-protective effect of maternal IgG specific to such antigens diminishes rapidly after these antibodies wane [45], which is usually by 6 months [6,46–48]. Thus, malaria-specific maternal antibodies likely would not explain reduced malaria infections in children older than 6 months.

Breast milk has been hypothesized to provide protection against malaria in the breastfed infant. While breastfeeding can confer non-malaria specific benefits to the developing infant immune system [49,50], evidence that breast milk derived maternal antimalarial antibodies protects against malaria infection of clinical malaria is lacking [5]. In mice, maternal IgG from breast milk can be transferred from the gut lumen to serum of pups via neonatal Fc receptors [51]. However, in humans, a recent case series of mothers given therapeutic monoclonal IgG1 antibodies for chronic inflammatory rheumatic conditions revealed minimal transfer of these antibodies to breastmilk with undetectable levels in the blood of their breastfed infants [52]. It must be noted that the WHO-recommended practice of exclusive breast feeding in infants under 6 months of age ranges widely from 31% to 84% across sub-Saharan African countries [53,54], with the majority of mothers stopping breastfeeding their child all together by 24 months of age [55]. Thus, any effect of breastfeeding on reducing malaria in young children would be geographically variable.

## Evidence for malaria-protective immune responses in young children

Despite being immature, the host immune response early in life can potentially thwart malaria infection at the pre-erythrocytic stage, control parasitemia and disease after establishment of blood-stage infection, or prime adaptive responses. Mosquito bites can provoke innate immune responses and affect T cell responses [56,57] which have been associated with reduced parasite burden in mouse models [58], but there is little evidence that host responses to bites by *Anopheles* mosquitoes can lead to protection against either malaria infection or clinical malaria in human studies. The children described as ‘malaria-resistant’ in the aforementioned Tanzanian birth cohort study by Nash and colleagues had higher levels of pro-inflammatory cytokines, particularly IL-1β, in the cord blood compared with susceptible children [15]. In another prospective study conducted in Benin, TLR-mediated IL-10 responses from whole blood obtained at birth were associated with increased risk of *P. falciparum* infection [59]. A birth cohort study of mother-infant pairs in Burkina Faso showed a mixed picture in terms of the association of inflammatory cytokine responses of TLR-agonist stimulated cord blood and malaria risk during the first year of life [60]. Note that studies that used cord blood or samples obtained at birth may be confounded by prenatal exposure to *P. falciparum* (i.e. placental malaria), which not only affects the neonatal immune response [7] but is also associated with increased risk of malaria in the first years of life that is itself confounded by relative differences in malaria exposure between mother-infant pairs [61–63]. Despite this caveat, the limited evidence to date seems to suggest that pro-inflammatory responses detected at birth are modestly associated with protection from *P. falciparum* infection but not necessarily clinical malaria once infected.

Recent studies of immune responses assessed after birth but early in life (<5 years) have provided further insight into potential roles of innate and early adaptive immunity in mitigating malaria risk. IgM antibodies to the α-gal glycan, which is expressed by pathogens including *Plasmodium* spp. and higher organisms except for birds, fish, and humans, can specifically inhibit hepatocyte invasion by *Plasmodium* sporozoites and have been shown to have an association with protection from clinical malaria, but not *P. falciparum* infection, in infants [64,65]. Higher production of pro-inflammatory cytokines, particular TNF, by *P. falciparum*-stimulated peripheral blood mononuclear cells isolated from Mozambican children up to 2 years of age was associated with reduced incidence of clinical malaria during the following 2 years of follow up [66]. In a systems vaccinology analysis of the PfSPZ malaria vaccine trial conducted in Kenyan infants mentioned above, we observed that innate immune activation and myeloid blood signatures at study baseline were associated with protection from *P. falciparum* parasitemia in placebo controls through 3 months of active and passive surveillance [13]. Despite being relatively immature, the malaria-specific antibody responses of very young children can control parasitemia and symptomatic malaria to some extent. IgM+ B cells specific for *P. falciparum* isolated from young Malian children aged 0–4 years have been shown to proliferate in response to acute malaria, and IgM isolated from these children can inhibit parasite growth *in vitro* [67]. In a longitudinal cohort of Papa New Guinean children aged 1–3 years, antibodies to PfEMP1 Group 2 DBLα variants were associated with reduced prospective risk of clinical malaria [68].

Age-dependent changes in circulating innate cells may partially explain the observed decreased risk of parasitemia in the youngest children. A subset of γδ T cells called Vγ9Vδ2^+^ T cells (or Vδ2^+^ T cells) respond to phosphoantigens produced by bacteria and parasites, including *Plasmodium*, and rapidly proliferate immediately after birth [69]. In a high-malaria transmission setting in Uganda, Vδ2^+^ T cell counts sharply decline after this initial expansion until ~4 years of age, but both the frequency and function of these Vδ2^+^ T cells were observed to correlate with protection from *P. falciparum* infection [70]. Additional studies are needed to confirm these findings and further assess whether other innate responses early in life correlate with reduced prospective risk of *P. falciparum* infection. However, such studies are difficult to conduct as they are resource intensive. Blood samples must be collected longitudinally at frequent intervals (e.g. every 2 weeks) followed by retrospective molecular analysis of samples to determine time-to-first infection (Figure 1A). Additionally, there are ethical and practical considerations that restrict the frequency and volume of blood obtained from young children. However, as multiomic technologies become more democratized, systems-based approaches would allow researchers to maximize the use of limited samples obtained from the youngest and most vulnerable children, potentially providing greater insight in the cellular immune response against the establishment of blood-stage infection early in life.

## The challenge of differentiating protection from infection versus lack of exposure in field studies

As noted in the Introduction, prospective field studies that define malaria protection as absence of *P. falciparum* infection or clinical malaria can potentially misclassify individuals as protected when they were either never exposed to infectious mosquito bites or malaria events were not fully captured due to insensitive detection or insufficient sampling [71]. For clinical episodes, this can be addressed by measuring the time from incident parasitemia to malarial symptoms in individuals with documented parasitemia and serves as a more reliable assessment of blood-stage immunity [47,72]. However, assessing pre-erythrocytic immunity in field studies poses additional challenges. In time-to-infection studies, not only can the non-malaria exposed be misclassified as protected, but the time-to-infection metric is, in essence, a time-to-detectable parasitemia metric and cannot distinguish between pre-erythrocytic and early blood-stage immunity [73]. In fact, it has been suggested that differences in time-to-detectable parasitemia can be largely attributable to acquisition of immunity that inhibits blood-stage growth [73]. Highly sensitive molecular detection of asexual parasites using larger blood volumes combined with more frequent sampling can address these issues to some extent but are resource intensive and thus impractical for large-scale field studies. A more scalable approach would be to confirm malaria exposure by measuring increases in antibodies specific for antigens from either *Anopheles* salivary glands [74] or the pre-erythrocytic or erythrocytic stages of the parasite [75,76] during the surveillance period. The application of down-selected protein microarrays containing hundreds of *P. falciparum* antigens from multiple stages would facilitate such studies [77,78].

## Future directions

The biology of malaria transmission, infection, and disease is undoubtedly complex given the *Plasmodium* parasite’s requirement for both mosquito and vertebrate hosts. Studying human immunity to the malaria infection in the field is made even more complex by the myriad of factors extrinsic to the host that may confound our interpretation of what constitutes protection against liver-stage infection, elite control of blood-stage growth, and lack of malaria exposure. The ‘aparasitemic’ phenotype described in this review can only be identified in prospective studies with high-resolution active infection surveillance, ideally using sensitive molecular detection of parasitemia. Such studies would require at least 200 children, as this unique phenotype represents only a small proportion of children when followed for more than a year, such was the case for the Tanzanian cohort noted above, where only 9.2% remained free of parasitemia [15]. Computational advances have allowed us to address complex biological questions using unbiased approaches that considers not only high-dimensional molecular and cellular features from the host’s peripheral blood (e.g. bulk and single-cell transcriptomics, metabolomics, functional antibody profiling) but also individual and population-level metadata such as exposure history and transmission intensity. Moving forward, integrated and comprehensive systems-based analyses that attempt to evaluate as many intrinsic and extrinsic features available would allow researchers to maximize the use of limited samples obtained from the youngest and most vulnerable children. This would potentially provide greater insight in the cellular immune response against the establishment of *P. falciparum* blood-stage infection early in life and generate new hypotheses that could be validated in other cohorts or experimentally using animal and *in vitro* cell culture models.

## Perspectives

- The observation that infants and young children living in settings of intense malaria transmission have less malaria infections than what would otherwise be expected for their level of immunity has long intrigued researchers.
- Although factors extrinsic to the young host’s immune system have been proposed as explanations for this observation, including reduced malaria exposure and maternally transferred immune factors, more recent evidence has suggested that innate and early adaptive immune responses contribute to protection from clinical malaria.
- Moving forward, unbiased systems-based approaches would maximize the use of limited samples obtained from the youngest and most vulnerable children, potentially provide greater insight in the cellular immune response against the establishment of *P. falciparum* blood-stage infection early in life.

## Abbreviations

EIR: entomological inoculation rate
PfSPZ: *P. falciparum* sporozoite
RDT: rapid diagnostic tests
